# Coronary artery bypass grafting outcomes of patients with human immunodeficiency virus: a population-based study of National Inpatient Sample from 2015 to 2020

**DOI:** 10.1038/s41598-024-65518-y

**Published:** 2024-06-22

**Authors:** Renxi Li, Deyanira J. Prastein, Brian G. Choi

**Affiliations:** 1 The George Washington University School of Medicine and Health Sciences, 2300 I St NW, Washington, D.C., 20052 USA; 2 Department of Surgery, The George Washington University Hospital, Washington, D.C., USA

**Keywords:** Human immunodeficiency virus, Acquired immune deficiency syndrome, Coronary artery bypass grafting, Risk factors, Mortality, Cardiology, Diseases, Health care, Medical research, Risk factors

## Abstract

Individuals affected by human immunodeficiency virus (HIV) have a growing demand for coronary artery bypass grafting (CABG) due to heightened risk for cardiovascular diseases and extended life expectancy. However, CABG outcomes in HIV patients are not well-established, with insights only from small case series studies. This study conducted a comprehensive, population-based examination of in-hospital CABG outcomes in HIV patients. Patients underwent CABG were identified in National Inpatient Sample from Q4 2015–2020. Patients with age < 18 years and concomitant procedures were excluded. A 1:5 propensity-score matching was used to address preoperative group differences. Among patients who underwent CABG, 613 (0.36%) had HIV and were matched to 3119 out of 167,569 non-HIV patients. For selected HIV patients, CABG is relatively safe, presenting largely similar outcomes. After matching, HIV and non-HIV patients had comparable in-hospital mortality rates (2.13% vs. 1.67%, *p* = 0.40). Risk factors associated with mortality among HIV patients included previous CABG (aOR = 14.32, *p* = 0.01), chronic pulmonary disease (aOR = 8.24, *p* < 0.01), advanced renal failure (aOR = 7.49, *p* = 0.01), and peripheral vascular disease (aOR = 6.92, *p* = 0.01), which can be used for preoperative risk stratification. While HIV patients had higher acute kidney injury (AKI; 26.77% vs. 21.77%, *p* = 0.01) and infection (8.21% vs. 4.18%, *p* < 0.01), other complications were comparable between the groups.

## Introduction

Coronary artery disease (CAD) affects nearly 16.5 million Americans, making it a leading cause of death in the US^1^. Coronary artery bypass grafting (CABG) stands as a widely-used coronary revascularization procedure, with over 200,000 procedures conducted annually in the US^2^.

Although the prevalence of human immunodeficiency virus (HIV) infection has risen in recent years, with the advent of effective antiretroviral therapy, the prognosis of individuals living with HIV has significantly improved, transforming HIV into a chronic condition for many patients^3^. Patients with HIV often have heightened proinflammatory responses and increased platelet reactivity, predisposing them to atherosclerosis development^4^. Additionally, antiretroviral therapy can elevate the risk of hypertension, hyperlipidemia, and metabolic syndrome, all of which are risk factors for cardiovascular diseases^5–8^. Consequently, given the extended life expectancy and heightened risk profiles, individuals with HIV face an elevated risk for CAD, leading to a growing demand for CABG procedures. Indeed, a prior study indicated a significant increase in the prevalence of HIV among cardiac surgery patients, with the majority undergoing CABG^9^.

The outcomes of CABG in patients with HIV are not well-established. Several small case series studies using institutional data have not identified HIV as a risk factor for mortality and complications post-CABG, but their conclusions are constrained by the limited sample sizes^10–12^. While some larger-scale studies utilizing the National/Nationwide Inpatient Sample (NIS) databases have explored cardiac surgery outcomes in HIV patients, all these studies did not distinguish CABG from other cardiac procedures^8,9,13^. This lack of distinction is likely attributed to the relatively small cohort of HIV patients in these studies, limiting the applicability of their findings directly to patients undergoing CABG.

This study aimed to conduct a comprehensive population-based analysis of the short-term outcomes of CABG among patients affected by HIV. Additionally, it aimed to identify risk factors associated with mortality in HIV patients after CABG. Consequently, the findings from this research could offer valuable insights for preoperative risk stratification and management of complications in HIV patients undergoing CABG.

## Methods

### Data source

The unweighted NIS database from the last quarter of 2015–2020 was used to identify patients who underwent CABG via the International Classification of Diseases, 10th Revision, Procedure Coding System (ICD-10-PCS) codes of 0210xxx. Patients who had concomitant aortic valve replacement (ICD-10-PCS 02RFxxx) and mitral valve replacement (ICD-10-PCS 02RGxxx), as well as those below 18 years of age, were excluded. Patients with HIV were identified by Elixhauser measure with International Classification of Diseases, Tenth Revision, Clinical Modification (ICD10-CM) codes of B20, O98.71, O98.72, O98.73, Z21^14^. Patients with and without HIV were stratified into the two study cohorts.

### Preoperative variables

Preoperative variables were compared between patients with and without HIV as shown in Tables 1 and 2. Table 1 includes patients’ sex, age, race and ethnicity, socioeconomic status, primary payer status, hospital characteristics, transfer status, and admission status. The average household income from the patient’s ZIP code was estimated and then used to stratify the patients into four quartiles based on the income of their neighborhood. Hospital characteristics included hospital bed size, location, and teaching status. Hospital bed sizes were stratified into small, medium, and large based on the American Hospital Association’s yearly survey of hospitals as well as the hospital’s location and teaching status. Table 2 includes the patient’s comorbidities and relevant diagnoses. Elixhauser comorbidities are a set of common comorbidities measured in administrative databases^14^. The patient’s comorbidities were identified by Elixhauser measure as well as by additional ICD-10-CM codes as listed in Table S1^14^.

**Table 1:** Comparing demographic, socioeconomic status, primary payer status, hospital characteristics, transfer status, and admission status between patients with and without HIV who underwent CABG before and after 1:5 propensity-score matching.

	HIV (n = 613)	Pre-match		Post-match	
		Non-HIV (n = 167,569)	*p*-value	Non-HIV (n = 3119)	*p*-value
Sex					
Males	531 (86.62%)	126,845 (75.7%)	< 0.01	2687 (86.4%)	1.00
Females	82 (13.38%)	40,710 (24.29%)	< 0.01	423 (13.6%)	1.00
Age					
Age < 55 years	166 (27.08%)	22,898 (13.66%)	< 0.01	848 (27.27%)	0.96
55 ≤ Age < 65 years	283 (46.17%)	48,587 (29%)	< 0.01	1416 (45.53%)	0.76
65 ≤ Age < 75 years	144 (23.49%)	62,634 (37.38%)	< 0.01	722 (23.22%)	0.96
75 ≤ Age < 85 years	19 (3.1%)	30,728 (18.34%)	< 0.01	118 (3.79%)	0.48
Age ≥ 85 years	1 (0.16%)	2722 (1.62%)	< 0.01	6 (0.19%)	1.00
Race and ethnicity					
Caucasian	323 (52.69%)	125,660 (74.99%)	< 0.01	1638 (52.67%)	0.93
African American	171 (27.9%)	11,525 (6.88%)	< 0.01	857 (27.56%)	0.80
Hispanic	65 (10.6%)	12,747 (7.61%)	0.01	330 (10.61%)	0.94
Asian	4 (0.65%)	5482 (3.27%)	< 0.01	18 (0.58%)	0.77
Native Americans	4 (0.65%)	901 (0.54%)	0.58	19 (0.61%)	0.78
Other races	27 (4.4%)	4866 (2.9%)	0.04	132 (4.24%)	0.83
Socioeconomic status					
Income 1st quartile (0–25%)	220 (35.89%)	46,001 (27.45%)	< 0.01	1137 (36.56%)	0.75
Income 2nd quartile (25–50%)	148 (24.14%)	45,341 (27.06%)	0.11	741 (23.83%)	0.88
Income 3rd quartile (50–75%)	109 (17.78%)	40,407 (24.11%)	< 0.01	529 (17.01%)	0.68
Income 4th quartile (75–100%)	101 (16.48%)	32,962 (19.67%)	0.04	529 (17.01%)	0.86
Primary payer status					
Medicare	295 (48.12%)	91,377 (54.53%)	< 0.01	1502 (48.3%)	0.82
Medicaid	77 (12.56%)	13,061 (7.79%)	< 0.01	421 (13.54%)	0.60
Private insurance	210 (34.26%)	52,175 (31.14%)	0.10	1035 (33.28%)	0.57
Self-pay	14 (2.28%)	4841 (2.89%)	0.47	81 (2.6%)	0.78
No charge	2 (0.33%)	521 (0.31%)	0.72	8 (0.26%)	0.67
Other payment	14 (2.28%)	5346 (3.19%)	0.25	61 (1.96%)	0.53
Hospital characteristics					
Rural hospital	9 (1.47%)	4579 (2.73%)	0.06	50 (1.61%)	1.00
Urban private practice	60 (9.79%)	26,337 (15.72%)	< 0.01	312 (10.03%)	0.88
Urban teaching hospital	544 (88.74%)	136,653 (81.55%)	< 0.01	2748 (88.36%)	0.78
Small bed size	50 (8.16%)	19,154 (11.43%)	0.01	240 (7.72%)	0.68
Medium bed size	164 (26.75%)	44,628 (26.63%)	0.96	785 (25.24%)	0.48
Large bed size	399 (65.09%)	103,787 (61.94%)	0.11	2085 (67.04%)	0.37
Transfer status					
No transfer in	503 (82.59%)	129,252 (77.13%)	< 0.01	2549 (81.96%)	0.73
Transferred in from a different acute care hospital	90 (14.78%)	34,002 (20.29%)	< 0.01	478 (15.37%)	0.76
Transferred in from another type of health facility	14 (2.3%)	3671 (2.19%)	0.68	71 (2.28%)	1.00
Admission status					
Elective	243 (39.9%)	77,181 (46.21%)	< 0.01	1268 (40.78%)	0.72
Emergent	366 (59.71%)	89,847 (53.62%)	< 0.01	1841 (59.2%)	0.69

CABG, coronary artery bypass grafting; HIV, human immunodeficiency virus.

**Table 2:** A comparison of comorbidities and relevant diagnoses between patients with and without HIV who underwent CABG before and after 1:5 propensity-score matching.

	HIV (n = 613)	Pre-match		Post-match	
		Non-HIV (n = 167,569)	*p*-value	Non-HIV (n = 3,119)	*p*-value
Comorbidities and relevant diagnoses					
Alcohol abuse	22 (3.59%)	5672 (3.38%)	0.74	94 (3.02%)	0.61
Autoimmune conditions	13 (2.12%)	4198 (2.51%)	0.70	65 (2.09%)	0.88
Lymphoma	2 (0.33%)	590 (0.35%)	1.00	7 (0.23%)	0.65
Leukemia	1 (0.16%)	697 (0.42%)	0.53	4 (0.13%)	0.59
Metastatic cancer	1 (0.16%)	381 (0.23%)	1.00	6 (0.19%)	1.00
Solid tumor without metastasis, in situ	0 (0%)	40 (0.02%)	1.00	0 (0%)	NA
Solid tumor without metastasis, malignant	10 (1.63%)	2170 (1.29%)	0.47	62 (1.99%)	0.75
Cerebrovascular disease	13 (2.12%)	3317 (1.98%)	0.77	51 (1.64%)	0.39
Heart failure	0 (0%)	140 (0.08%)	1.00	0 (0%)	NA
Dementia	1 (0.16%)	1778 (1.06%)	0.03	12 (0.39%)	0.71
Depression	107 (17.46%)	14,776 (8.82%)	< 0.01	562 (18.07%)	0.82
Diabetes without chronic complications	77 (12.56%)	28,198 (16.83%)	< 0.01	415 (13.34%)	0.69
Diabetes with chronic complications	196 (31.97%)	54,049 (32.25%)	0.90	1004 (32.28%)	1.00
Drug abuse	45 (7.34%)	2918 (1.74%)	< 0.01	212 (6.82%)	0.60
Complicated hypertension	280 (45.68%)	60,037 (35.83%)	< 0.01	1403 (45.11%)	0.76
Uncomplicated hypertension	252 (41.11%)	87,135 (52%)	< 0.01	1306 (41.99%)	0.69
Moderate to advanced liver disease	0 (0%)	144 (0.09%)	1.00	0 (0%)	NA
Chronic pulmonary disease	131 (21.37%)	36,308 (21.67%)	0.88	686 (22.06%)	0.75
Obesity	106 (17.29%)	48,764 (29.1%)	< 0.01	564 (18.14%)	0.69
Paralysis	8 (1.31%)	2382 (1.42%)	1.00	23 (0.74%)	0.15
Peripheral vascular disease	72 (11.75%)	23,628 (14.1%)	0.10	373 (11.99%)	0.89
Advanced renal failure	35 (5.71%)	3990 (2.38%)	< 0.01	171 (5.5%)	0.77
Hypothyroidism	43 (7.01%)	18,477 (11.03%)	< 0.01	190 (6.11%)	0.36
Other thyroid disorders	6 (0.98%)	1717 (1.02%)	1.00	30 (0.96%)	1.00
Valvular diseases	3 (0.49%)	873 (0.52%)	1.00	13 (0.42%)	0.74
Left ventricle dysfunction	3 (0.49%)	470 (0.28%)	0.25	16 (0.51%)	1.00
Pulmonary hypertension	27 (4.4%)	7527 (4.49%)	1.00	151 (4.86%)	0.76
Endocarditis	4 (0.65%)	187 (0.11%)	0.01	16 (0.51%)	0.56
Nonrheumatic mitral valve disorders	49 (7.99%)	12,931 (7.72%)	0.76	229 (7.36%)	0.56
Nonrheumatic aortic valve disorders	17 (2.77%)	6684 (3.99%)	0.15	87 (2.8%)	1.00
Nonrheumatic tricuspid valve disorders	3 (0.49%)	1246 (0.74%)	0.64	11 (0.35%)	0.49
Nonrheumatic pulmonary valve disorders	2 (0.33%)	275 (0.16%)	0.27	6 (0.19%)	0.63
Atrial fibrillation	140 (22.84%)	56,447 (33.69%)	< 0.01	724 (23.28%)	0.83
Atrial flutter	34 (5.55%)	7184 (4.29%)	0.13	173 (5.56%)	1.00
Ventricular fibrillation	19 (3.1%)	3420 (2.04%)	0.08	93 (2.99%)	0.90
Ventricular flutter	0 (0%)	29 (0.02%)	1.00	0 (0%)	NA
Sick sinus syndrome	0 (0%)	2301 (1.37%)	< 0.01	2 (0.06%)	1.00
First-degree atrioventricular block	4 (0.65%)	2411 (1.44%)	0.12	24 (0.77%)	1.00
Second-degree atrioventricular block	0 (0%)	1004 (0.6%)	0.06	0 (0.00%)	NA
Complete atrioventricular block	5 (0.82%)	2032 (1.21%)	0.46	25 (0.8%)	1.00
Carotid artery disease	1 (0.16%)	331 (0.2%)	1.00	4 (0.13%)	0.59
Hyperlipidemia	478 (77.98%)	135,575 (80.91%)	0.07	2445 (78.62%)	0.79
Anemia	51 (8.32%)	7499 (4.48%)	< 0.01	240 (7.72%)	0.56
Thrombocytopenia	114 (18.6%)	32,076 (19.14%)	0.76	595 (19.13%)	0.87
Sleep apnea	61 (9.95%)	26,207 (15.64%)	< 0.01	313 (10.06%)	0.94
Tobacco use	343 (55.95%)	84,943 (50.69%)	0.01	1758 (56.53%)	0.89
Previous MI	148 (24.14%)	30,473 (18.19%)	< 0.01	759 (24.41%)	0.84
Previous CVA	112 (18.27%)	29,036 (17.33%)	0.52	583 (18.75%)	0.65
Previous CABG	20 (3.26%)	3645 (2.18%)	0.07	106 (3.41%)	1.00
Previous PCI	40 (6.53%)	11,883 (7.09%)	0.64	206 (6.62%)	0.93
Previous valve replacement	1 (0.16%)	513 (0.31%)	1.00	6 (0.19%)	1.00

CABG, coronary artery bypass grafting; CVA, cerebrovascular accident; HIV, human immunodeficiency virus; MI, myocardial infarction; NA, not applicable; PCI, percutaneous coronary intervention.

### Postoperative variables

In-hospital outcomes after CABG were examined (Table 3). The outcomes included mortality, major adverse cardiovascular event (MACE), myocardial infarction (MI), stroke, transient ischemic attack (TIA), neurological complications, pericardial complications, pacemaker implantation, cardiogenic shock, respiratory complications, mechanical ventilation, acute kidney injury (AKI), post-procedural renal failure, venous thromboembolism (VTE), pulmonary embolism (PE), hemorrhage/hematoma, infection, sepsis, deep wound complication, superficial wound complication, vascular complication, diaphragmatic paralysis, and reopen surgery for bleeding control. MACE was defined as MI, stroke, postprocedural, cardiogenic shock, postprocedural heart failure, and/or postprocedural cardiac insufficiency. In addition, transfer out rate, time from admission to operation, hospital length of stay (LOS), and total hospital charge were examined. The ICD10-CM/PCS codes were used to define these outcomes, as listed in Table S2.

**Table 3:** Comparison of in-hospital outcomes between patients with and without HIV who underwent CABG after 1:5 propensity-score matching.

	HIV (n = 613)	Non-HIV (n = 3,119)	*p*-value
Mortality	13 (2.13%)	52 (1.67%)	0.40
MACE	15 (2.46%)	96 (3.09%)	0.51
MI	9 (1.48%)	62 (1.99%)	0.52
Stroke	2 (0.33%)	19 (0.61%)	0.56
TIA	0 (0%)	8 (0.26%)	0.37
Neurological complications	3 (0.49%)	27 (0.87%)	0.46
Pericardial complications	10 (1.64%)	41 (1.32%)	0.57
Pacemaker implantation	5 (0.82%)	26 (0.84%)	1.00
Cardiogenic shock	0 (0%)	0 (0%)	NA
Respiratory complications	72 (11.82%)	354 (11.38%)	0.78
Mechanical ventilation	69 (11.33%)	278 (8.94%)	0.07
AKI	163 (26.77%)	677 (21.77%)	0.01
Post-procedural renal failure	2 (0.33%)	23 (0.74%)	0.41
VTE	1 (0.16%)	18 (0.58%)	0.35
PE	0 (0%)	1 (0.03%)	1.00
Hemorrhage/hematoma	351 (57.64%)	1728 (55.56%)	0.35
Infection	50 (8.21%)	130 (4.18%)	< 0.01
Sepsis	0 (0%)	1 (0.03%)	1.00
Deep wound complication	0 (0%)	6 (0.19%)	0.60
Superficial wound complication	6 (0.99%)	18 (0.58%)	0.27
Vascular complication	1 (0.16%)	25 (0.8%)	0.11
Diaphragmatic paralysis	1 (0.16%)	4 (0.13%)	0.59
Reopen surgery	3 (0.49%)	15 (0.48%)	1.00
Transfer out	119 (19.54%)	506 (16.27%)	0.04

	Mean ± SD	Mean ± SD	*p*-value
Admission to operation (days)	2.96 ± 3.64	2.75 ± 3.23	0.22
LOS (days)	10.41 ± 7.65	10.03 ± 7.59	0.26
Total hospital charge (US dollars)	261,011 ± 337,816	230,783 ± 241,233	0.04

AKI, acute kidney injury; CABG, coronary artery bypass grafting; HIV, human immunodeficiency virus; LOS, length of stay; MACE, major adverse cardiovascular event; MI, myocardial infarction; NA, not applicable; PE, pulmonary embolism; SD, standard deviation; TIA, transient ischemic attack; VTE, venous thromboembolism.

### Statistical analysis

Fisher’s exact test was used to compare the pre-operative variables between patients with and without HIV. To account for the preoperative differences between the HIV and non-HIV cohorts as well as their significant differences in sample sizes, a propensity-score matching was conducted between the two groups in a 1:5 ratio (HIV: non-HIV) using the Greedy Matching algorithm with a 2% caliper. After the matching, Fisher’s exact test was used to compare postoperative outcomes that were binary. Two-tailed independent t-tests were used to compare continuous outcomes.

In addition, among HIV patients, risk factors associated with in-hospital mortality were identified by a multivariable logistic regression model with stepwise backward selection. Multicollinearity tests were then used to confirm the independency of the identified risk factors, indicated by tolerance > 0.20 and condition index < 10.

All statistical analyses were conducted using SAS, version 9.4. A *p*-value less than 0.05 was defined as statistically significant. The authors had full access to the dataset and took responsibility for the integrity of all analyses. Given this retrospective study used a de-identified NIS dataset, the study was not considered a human-subject study and was exempted from Institutional Review Board (IRB) review at The George Washington University. Informed patient consent was therefore not required. All methods were performed in accordance with the relevant guidelines and regulations of The George Washington University.

### Ethics approval

This study was exempt from the IRB approval by The George Washington University as it analyzed retrospective, deidentified NIS data.

## Results

Between the last quarter of 2015 and 2020, 613 (0.36%) patients with HIV underwent CABG, who were matched to 3,119 out of 167,569 patients who did not have HIV.

A comparison between demographic, socioeconomic status, primary payer status, hospital characteristics, transfer status, and admission status between patients with and without HIV who underwent CABG is shown in Table 1. Patients with HIV were more likely to be males (86.62% vs. 75.70%, *p* < 0.01), have age less than 65 years (55–65 years, 46.17% vs. 29.00%, *p* < 0.01; less than 55 years, 27.08% vs. 13.66%, *p* < 0.01), be African American (27.90% vs. 6.88%, *p* < 0.01), Hispanic (10.60% vs. 7.61%, *p* = 0.01), or other races (4.40% vs. 2.90%, *p* = 0.04), have income at the lowest quartile (0–25%) (35.89% vs. 27.45%, *p* < 0.01), under Medicaid (12.56% vs. 7.79%, *p* < 0.01), stay in an urban teaching hospital (88.74% vs 81.55%, *p* < 0.01), have no transfer in/directly admitted (82.59% vs. 77.13%, *p* < 0.01), and under emergent admission (59.71% vs. 53.62%, *p* < 0.01). In contrast, patients with HIV were less likely to have age over 65 years (65–75 years, 23.49% vs. 37.38%, *p* < 0.01; 75–85 years, 3.1% vs. 18.34%, *p* < 0.01; over 85 years, 0.16% vs. 1.62%, *p* < 0.01), be Caucasian (52.69% vs. 74.99%, *p* < 0.01) or Asian (0.65% vs. 3.27%, *p* < 0.01), have income at the 3rd quartile (50–75%) (17.78% vs. 24.11%, *p* < 0.01) or the 4th quartile (75–100%) (16.48% vs. 19.67%, *p* = 0.04), use Medicare (48.12% vs. 54.53%, *p* < 0.01), stay in an urban private practice (9.79% vs. 15.72%, *p* < 0.01) or a hospital with small bed size (8.16% vs. 11.43%, *p* = 0.01), transferred in from a different acute care hospital (14.78% vs. 20.29%, *p* < 0.01), or under elective admission (39.90% vs. 46.21%, *p* < 0.01). All differences were addressed by the 1:5 propensity-score matching.

Comorbidities and relevant diagnoses between patients with and without HIV who underwent CABG are summarized in Table 2. Patients with HIV were more likely to have depression (17.46% vs. 8.82%, *p* < 0.01), drug abuse (7.34% vs. 1.74%, *p* < 0.01), complicated hypertension (45.68% vs. 35.83%, *p* < 0.01), advanced renal failure (5.71% vs. 2.38%, *p* < 0.01), endocarditis (0.65% vs. 0.11%, *p* = 0.01), anemia (8.32% vs. 4.48%, *p* < 0.01), tobacco use (55.95% vs. 50.69%, *p* = 0.01), or previous myocardial infarction (MI; 24.14% vs. 18.19%, *p* < 0.01). On the other hand, patients with HIV were less likely to have dementia (0.16% vs. 1.06, *p* = 0.03), diabetes without chronic complications (12.56% vs. 16.83%, *p* < 0.01), uncomplicated hypertension (41.11% vs. 52.00%, *p* < 0.01), obesity (17.29% vs. 29.10%, *p* < 0.01), hypothyroidism (7.01% vs. 11.03%, *p* < 0.01), atrial fibrillation (22.84% vs. 33.69%, *p* < 0.01), sick sinus syndrome (0.00% vs. 1.37%, *p* < 0.01), or sleep apnea (9.95% vs. 15.64%, *p* < 0.01). The differences in comorbidities and relevant diagnoses were matched by the 1:5 propensity-score matching.

The in-hospital outcomes between patients with and without HIV who underwent CABG were summarized in Table 3. After 1:5 propensity-score matching, HIV and non-HIV patients had comparable mortality rates (2.13% vs. 1.67%, *p* = 0.40). However, patients with HIV were more likely to have AKI (26.77% vs. 21.77%, *p* = 0.01) and infection (8.21% vs. 4.18%, *p* < 0.01). Also, HIV patients had higher rates of transferring out (19.54% vs. 16.27%, *p* = 0.04) and higher total hospital charges (261,011 ± 337,816 vs. 230,783 ± 241,233 US dollars, *p* = 0.04). Other risks of complications, time from admission to operation, and LOS were all comparable between the two groups.

The risk factors associated with in-hospital mortality among HIV patients are shown in Table 4. The risk factors included previous CABG (aOR 14.32, 95% CI 2.24–91.63, p = 0.01), chronic pulmonary disease (aOR 8.24, 95% CI 1.91–35.58, *p* < 0.01), advanced renal failure (aOR 7.49, 95% CI 1.53–36.79, *p* = 0.01), and peripheral vascular disease (aOR 6.92, 95% CI 1.64–29.25, *p* = 0.01). Multicollinearity tests on all identified variables showed tolerance > 0.20 and condition index < 10, indicating independency between the risk factors.

**Table 4:** Risk factors associated with in-hospital mortality among patients with HIV who underwent CABG.

Risk factors	aOR	95% CI	*p*-value
Previous CABG	14.32	2.24–91.63	0.01
Chronic pulmonary disease	8.24	1.91–35.58	< 0.01
Advanced renal failure	7.49	1.53–36.79	0.01
Peripheral vascular disease	6.92	1.64–29.25	0.01

aOR, adjusted odds ratio; CI, confidence interval; CABG, coronary artery bypass grafting; HIV, human immunodeficiency virus.

## Discussion

This study conducted a population-based analysis of the in-hospital outcomes of CABG among patients affected by HIV. Patients with HIV had more demographic/socioeconomic disparities and comorbid burdens. After propensity-score matching, patients with HIV had higher AKI and infection, as well as higher transfer rates and hospital charges. However, mortality and other complications were comparable between HIV and non-HIV patients. Risks associated with in-hospital mortality among HIV patients included previous CABG, chronic pulmonary disease, advanced renal failure, and peripheral vascular disease.

In the US, approximately 1.2 million individuals, representing about 0.3% of the population, are living with HIV^15^. While care for individuals with HIV has received increased attention, research on CABG outcomes for this group remains very limited, likely due to their low representation in the population which constrains meaningful analysis with adequate statistical power. For instance, previous studies have shown that the prevalence of HIV among those undergoing any cardiac surgery was a mere 0.2%^8,9^. However, Polanco et al. revealed that the proportion of HIV patients in cardiac surgeries doubled from 0.1 to 0.2% between 2000 and 2010, likely due to the expansion of effective antiviral treatment^9^. While a previous study found HIV patients to be less likely to undergo CABG^13^, this study, which used the latest NIS dataset from 2015 to 2020, found the prevalence of HIV among CABG patients to be as high as 0.36%. This significant difference in representation may suggest an ongoing increase in the accessibility of CABG for HIV patients, aligning with the trend found by Polanco et al.^9^. However, it is also worth considering that the use of the more granular ICD-10 coding system in this study could offer better identification of HIV patients compared to the ICD-9 system used in previous NIS studies^8,9^.

Patients with HIV have an increased risk of developing cardiovascular diseases by 50–100%^16^. This elevated risk can be attributed to inherent HIV-related factors such as chronic inflammation and platelet dysfunction as well as side effects from antiretroviral therapies, including hypertension, hyperlipidemia, and metabolic syndrome^4–8^. For instance, our study revealed a markedly higher prevalence of complicated hypertension among HIV patients by almost one-third. Furthermore, this study found demographic and socioeconomic disparities that might be linked to the development of CAD in HIV-positive individuals. HIV patients were more likely to be African Americans or come from lower-income neighborhoods, both of which have been recognized as independent risk factors for CAD^17–19^. Additionally, the HIV cohort in this study exhibited higher rates of drug abuse and tobacco usage, which are well-established contributors to cardiovascular diseases^20–22^.

Previous small case series studies did not find any differences in short- or mid-term mortality rates between HIV and non-HIV patients undergoing CABG^10–12^. However, these conclusions can be significantly constrained by the small sample sizes, which range from only 5 to 26 HIV patients^10–12^. Larger-scale national registry studies also found no disparities in in-hospital mortality following cardiac surgeries for both cohorts, but these studies did not differentiate CABG from other cardiac procedures^8,9,13^. This study aligns with these findings, noting no significant mortality differences between the HIV and non-HIV groups. However, the unique strength of this study lies in its expansive, population-based dataset. This allowed for the identification of risk factors associated with in-hospital mortality in HIV patients, which can enhance pre-operative risk stratification for these patients set to undergo CABG.

Although various case series studies found similar short- and mid-term morbidities after CABG for HIV and non-HIV patients^10–12^, Boccara et al. found HIV patients might be at a higher risk for long-term MACE, primarily due to an increased risk for repeated revascularization^10^. In the present study, most in-hospital complications, including MACE, were comparable between the HIV and non-HIV cohorts. However, HIV patients exhibited elevated risks for AKI and infections. The heightened infection risk among HIV patients after surgery is expected, given their compromised immune response. The predisposition of HIV patients to AKI can be attributed to factors such as immunodeficiency, immune system reconstitution, or the nephrotoxic effects associated with antiretroviral therapy^23–26^.

This study has several limitations to acknowledge. Firstly, the NIS, being an administrative database, does not record clinical data including CD4 counts, viral loads, or the usage of antiviral therapies in HIV patients. Also, several factors that can influence revascularization outcomes, such as ejection fraction, coronary segment, stenosis diameter, lesion presence, coronary artery dominance, and small vessel diffusion, are not recorded in the NIS^27,28^. The absence of the information in the NIS dataset also prevents the calculation of surgical risk calculators like the Society of Thoracic Surgeons (STS) or the European System for Cardiac Operative Risk Evaluation (EuroSCORE) scores. Additionally, the NIS database only has in-hospital outcomes with no follow-ups, limiting the analysis of long-term prognosis among HIV patients after CABG. Nevertheless, the NIS is the most comprehensive all-payer database in the US, accounting for 20% of nationwide hospital discharges. While other large-scale cardiac surgery databases, like the STS databases, provide more granular records, they do not allow the identification of patients with HIV. Hence, the NIS is likely to be one of the few sources with both the statistical power and enough details to investigate CABG outcomes in patients with HIV.

In conclusion, using the NIS database, this study offers a comprehensive, population-based examination of short-term CABG outcomes in HIV patients. For selected HIV patients, CABG is relatively safe, presenting largely similar outcomes with the exceptions of higher AKI and infection rates. Factors associated with in-hospital mortality among HIV patients include prior CABG, chronic pulmonary disease, advanced renal failure, and peripheral vascular disorders, which can enhance preoperative risk stratification for HIV patients. Future population-based research should further explore the long-term prognosis of CABG in HIV-affected individuals.

### Supplementary Information

Supplementary Tables.

## Data Availability

The data that support the findings of this study are available on request from the corresponding author.
