# Author Correction: Coronary artery bypass grafting outcomes of patients with human immunodeficiency virus: a population-based study of National Inpatient Sample from 2015 to 2020

**DOI:** 10.1038/s41598-024-68135-x

**Published:** 2024-08-01

**Authors:** Renxi Li, Deyanira J. Prastein, Brian G. Choi

**Affiliations:** 1 The George Washington University School of Medicine and Health Sciences, 2300 I St NW, Washington, D.C., 20052 USA; 2 Department of Surgery, The George Washington University Hospital, Washington, D.C., USA

Correction to: *Scientific Reports* 10.1038/s41598-024-65518-y, published online 22 June 2024

The original version of this Article contained an error in the Discussion section.

“In the US, approximately 120 million individuals, representing about 0.3% of the population, are living with HIV^15^.”

now reads:

“In the US, approximately 1.2 million individuals, representing about 0.3% of the population, are living with HIV^15^.”

The original Article has been corrected.

